# Progress in Medicine

**Published:** 1896-12-26

**Authors:** 


### Progress in Medicine.

### DISEASES OF THE NERVOUS SYSTEM.

**Anatomy and Physiology.**—The function of the nerve-cells has quite recently become a moot point. Is their function merely trophic or are they, as we have hitherto believed, an integral and essential part of the nerve centres as regards the generation and transmission of nervous impulses. F. X. Dercum[1] points out some of the difficulties in which we are landed, if we accept the new views advanced, more especially by Nansen, who maintains that the reflex arcs are constituted first by the centripetal nerve and its fibrillary ramifications passing directly into the meshwork of the cortex—that is, into the molecular layer; secondly, by the propagation of the excitation through this molecular plexus; thirdly, by the transmission of stimuli to the minute lateral branches of the centrifugal or motor nerve fibres. It follows that impulses are transmitted to the superior centres without passing directly through the nerve-cells. Similarly Nansen believes that the voluntary impulses emanating from the nerve fibres which emerge from the superior centres transmit themselves directly to the centrifugal fibres of the inferior centres without passing through the nerve-cells of these centres; and further, that it is impossible to admit that the nerve-cells of the inferior centres possess direct importance either in relation to reflex movements or to the voluntary movements, and that this seems to apply equally well to the nerve-cells of the superior centres. Dercum remarks that this view forces us to the conclusion that the activity of the nervous system, intelligence, consciousness, &c., is really seated in a fibrillary meshwork of the cortex, the molecular layer, and has nothing to do with the nerve-cells of the cortex. The latter, indeed, deprived of their psychic functions, become simply "trophic" centres. They serve only for the maintaining of the nutrition of the nerve fibres and their innumerable arborescent ramifications. Mills, in the "Text-book on Nervous Diseases by American Authors," has maintained this position; he holds that "impulses are conveyed from processes to processes through the entire reflex arc, through the entire length of a cortico-efferent, or a cortico-afferent, projection system," without passing through nerve-cells, and that "the function of the nerve-cell body is trophic;" that its "nuclei and nucleoli preside over the nutrition of the long or short fibres which pass out of or grow into them"; and, further, that cells are of enormous bulk in order that they may be able to sustain these processes. In his words: "The aggregations of grey matter at various levels of the nervous system are watering and feeding places, not places for renewing nerve activity." Dercum maintains that nowhere in the whole range of biology do we find a similar anomaly obtain as is implied by this view. It asserts that merely incidental structural attributes are of greater value than the individual cells, whose building up constitutes the tissue. Not only upon general principles is this view subversive of all that we have hitherto learned; but, if carefully analysed, it is found to present insuperable difficulties. If it be true that a nerve-fibre diffuses the energy which it conveys: in a general way, scatters it through all the fibres or nerve-cell processes near which it happens to lie, it becomes impossible to explain the definite and precise actions of the nervous system, properties which are so characteristic of it. Nothing but hopeless confusion of function could result if such a thing were possible. It would mean that nerve-currents course indiscriminately without relation to each other through this network of fibres. It would mean that everything had been done by nature to conserve and isolate nervous impulses by enclosing the nerve-fibres in special sheaths of insulating material previous to their entrance into the cortex had, after all, no purpose, because in the end the currents are turned wildly loose into a common receptacle. The conservation of nerve-currents along cell processes, no matter how long these may be, or whether they be in the cortex or below it, is an absolute requisite.

Such facts as we possess are directly opposed to a diffusion of nervous energy. According to Berkley, by far the larger number of the finer fibres of the cortex are medullated almost to the extremities of the end-terminations. It is highly probable, as Berkley maintains, "that in no instance except at the free termination is there actually such a thing as a naked axis-cylinder."

Far from lending support to the view of Nansen, that the cell is to be left out of account in the consideration of nervous action, the discoveries of Golgi, Ramon Cajal, Van Gehuchten, and others have shown exactly the reverse. They have demonstrated beyond all question that, as in all other tissues, the cell is the actual integral structure. And while it was formerly held that the various cell-processes anastomosed freely with each other, we now know as a matter of fact that such anastomoses do not occur. The nerve-cell is a cell entirely by itself. It is a cell as distinct and as self-limited as any cell of any tissue with which we are acquainted. Far from being continuous through its processes with other cells, we learn that its processes nowhere fuse with other structures. Its processes are well limited, sharply defined, and bear no relation to those of other cells save that of propinquity or, perhaps, contact. The individuality of the nerve-cell as a morphological integer is wholly preserved. If we grasp this idea in its full meaning, our conception of the nervous system changes profoundly. It is no longer a stringing together of telegraph wires and way stations, but it consists of an aggregation of cell-integers, each one of which does its share in the production and in the transmission of nervous energy. For instance, the impulse proceeding from a motor neuron in the cortex is transmitted by the neuron through its own protoplasmic extension (the efferent nerve-fibres) to a definite aggregation of cells in the spinal cord. It communicates its energy to these cells in the spinal cord without in any way fusing with their protoplasm or their processses.

A consideration of the above facts suggested to Dercum the following thought: Can it be that the neuron is not an absolutely fixed morphological element? Can it be that it possesses a certain, though perhaps limited, power of movement? He found that this thought had occurred independently to three observers, one in Germany and two in France.[3]

While Ramon Cajal[4] opposes the theory of mobility of the neuron, he maintains, on the other hand, that the neuroglia cells possess a great degree of mobility. Ramon Cajal maintains that the processes of the neuroglia cells in reality represent an insulating or non-conducting material, and that during the period of relaxation they penetrate between the arborisations of the nerve-cells and their protoplasmic processes, and render difficult or impossible the passage of nerve-currents; on the other hand, when the processes of the neuroglia cells are retracted, the various nerve-cell processes, which they formerly separated from each other, are now permitted to come into contact. It certainly does not matter whether the nerve-cell processes move little or move much, but that they move at all is the question at issue; and this, it seems to Dercum, Ramon Cajal admits, although he makes that movement a purely passive one, and dependent upon the interposition of the processes of the neuroglia-corpuscles. It is certainly a minor point whether the movement of the nerve-cell processes is active or passive, But a single positive observation outweighs all other negative observations, no matter how great the authority behind them, and this positive observation has actually been made. Wiedersheim[5] actually saw in the living animal, leptodora hyalina, an entomostracan, the nerve-cells in tho œsophageal ganglion move. The œsophageal ganglion may in one sense be regarded as the brain of the animal, inasmuch as it receives the fibres of the optic nerve; and Wiedersheim saw its cells move and change their shape. He describes their movement as slow and flowing. Even if the animal in which the phenomena were observed is far removed from the vertebrates, it must be remembered that it is just in the lower forms that general biological truths must be sought for, and it is just in the lower forms that they have been found. What a flood of light is cast upon the subject of hysteria; thus, taking the simple example of a hysterical paralysis, we see how easily it is explained. The neurons of a certain area of the cortex, for instance, retract the terminal branches of the neuraxon to such an extent that the latter are no longer in contact, or sufficiently near to the neurons of the spinal cord which supply the muscles of the paralysed part. It explains also the marvellous fact that a hysterical paralysis may at one time be so real, so genuine, as to be indistinguishable from a grossly organic paralysis, and yet the next moment, upon a suggestion, may absolutely disappear. The shifting of symptoms in hysteria, this sudden disappearance of paralysis or anæsthesia, can be explained by the view here advanced as it can be by no other. When power is suddenly re-established in a hysterically-palsied limb, it simply means that the terminal branches of the cortical neuraxon, previously retracted, are again extended, so as to re-establish the proper relations with the spinal neurons. Take, again, the example of a hysterical anæsthesia. How often do we see a segmented anæsthesia or a hemi-anæsthesia coming and going under the influence of no other stimulus than that which applies to the psychic make-up of the individual, namely, a treatment which we call mental or moral treatment, or that more powerful treatment, suggestion under hypnotism.

When we turn to hypnotism we can see what a ready explanation it affords for the phenomena presented. Under the fixed stare necessitated by the ordinary method of bringing about hypnosis, and under the suggestion of sleep, the neurons are thrown into certain fixed relations with each other, corresponding solely to the ocular strain and singleness of thought induced. At the same time such relations of the neurons as ordinarily bring them into true contact with the outer world are suspended, probably by retraction of cell processes. We can easily understand, in the light of the theory here advanced, how, under hypnotic suggestion, a hysterical paralysis disappears, or how, under hypnotic suggestion, anæsthesia is produced in this or that part of the body. Further, the various stages of hypnotism itself—lethargy, catalepsy, somnambulism—are all of them capable of a scientific explanation upon this theory. In somnambulism again, certain of the neurons, especially those which stand in direct relation with the various sensory organs, form partial combinations with a limited number of other cortical neurons, so as to produce the various limited physic phenomena characteristic of somnambulism, whilst the great bulk of the neurons of the cortex, the summation of whose action constitutes the ego, and brings it into close relation with the outer world, have their processes retracted in sleep.

Leaving this interesting field, let us see for a moment of what enormous value this interpretation of cortical action is for normal mental phenomena. Sleep, instead of resulting from brain anæmia, or some other apocryphal condition of the circulation, merely means that when the substance of the cortical cells has been diminished by functional activity, which diminution we have reason to infer from the researches of Hodge,[6] there comes a time when the cell-processes are retracted, so that the neurons no longer stand in active relation to each other.

Many other thoughts also suggest themselves, and some are extremely interesting. For instance, the receptivity and marvellous adaptability of youth and early manhood appear to depend upon the relatively great mobility of cell-processes, while the retarded and restricted mental action so often seen in old age appears to be related to a much-diminished mobility of these processes. Dercum's communication, which is full of original conception, offers most valuable criticism on one of the moot points in practical neurology, and we have consequently extracted the paper somewhat fully.

[1] Amer. Journ. of Med. Sci., Aug., 1896. [2] Med. News, Nov. 9, p. 506. [3] Rabl.-Ruckhard. Neurol. Central, 1890; Lepine, Rev. de Med., Aug. 1894; Duval, Commun. to Soc. de Biol., 1895. [4] Rev. de Med. y Cir. Prac., May 9, 1895. [5] Anat. Auz., 1890, p. 675. [6] Journ. of Morph., Vol. vii., p. 95.

